# Effect of Mineral Trioxide Aggregate, Calcium-Enriched Mixture Cement and Mineral Trioxide Aggregate with Disodium Hydrogen Phosphate on BMP-2 Production 

**Published:** 2014-07-05

**Authors:** Negin Ghasemi, Saeed Rahimi, Mehrdad Lotfi, Jafar Solaimanirad, Shahriar Shahi, Hajar Shafaie, Amin Salem Milani, Sahar Shakuie, Vahid Zand, Majid Abdolrahimi

**Affiliations:** a Department of Endodontics, Dental Faculty, Tabriz University of Medical Sciences, Tabriz, Iran; b Dental and Periodontal Research Center, Department of Endodontics, Dental Faculty, Tabriz University of Medical Sciences, Tabriz, Iran; c Research Center for Pharmaceutical Nanotechnology and Department of Endodontics, Dental Faculty, Tabriz University of Medical Sciences, Tabriz, Iran; d Department of Anatomy and Histology, Faculty of Medicine, Tabriz University of Medical Sciences, Tabriz, Iran

**Keywords:** Bone Morphogenic Protein-2, Calcium-Enriched Mixture Cement, Disodium Hydrogen Phosphate, Fibroblasts, Mineral Trioxide Aggregate, Transforming Growth Factor-Beta

## Abstract

**Introduction:** One of the hypotheses regarding the calcification induction by mineral trioxide aggregate (MTA) is the involvement of transforming growth factor-Beta (TGF-β) super family. Calcium-enriched mixture (CEM) cement is one of the endodontic biomaterials with clinical applications similar to MTA. The aim of the present *in vitro* study was to compare the induction of bone morphogenic protein-2 (BMP-2) by a combination of disodium hydrogen phosphate (DSHP) and tooth colored ProRoot MTA (WMTA), to that of CEM cement and WMTA. **Methods and Materials:** Human gingival fibroblasts (HGFs) were obtained from the attached gingiva of human premolars. HGFs were cultured in Dulbecco’s Modified Eagle’s medium, supplemented with 10% fetal calf serum, penicillin, and streptomycin. Cells in groups 1, 2 and 3 were exposed to WMTA, CEM and WMTA+DSHP discs, respectively. The fourth group served as the control. After 72 h of exposure, HGF viability was determined by Mosmann’s tetrazolium toxicity (MTT) assay. BMP-2 levels in cell-free culture media were determined by enzyme-linked immunosorbent assay (ELISA). Statistical analysis was performed using the one-way ANOVA, followed by the post hoc Games-Howell test for BMP-2 and post hoc Tukey’s test for the results of MTT assay. **Results:** Cellular viability was significantly higher in group 3 compared to the other groups (*P*<0.05); however, CEM and WMTA did not exhibit significant differences (*P*=0.08). The control group exhibited significantly higher cellular viability in comparison to the other groups of the study (*P*<0.05). The highest and lowest protein production rates were observed in the WMTA (3167±274.46 pg/mL) and WMTA+DSHP (1796±839.49 pg/mL) groups, respectively. There were no significant differences between the control, WMTA and CEM groups (*P*>0.05). **Conclusion: **WMTA and CEM did not exhibit any significant differences in terms of inducing BMP-2 production; however, incorporation of DSHP into WMTA resulted in a decrease in the induction of this protein.

## Introduction

As an endodontic biomaterial, mineral trioxide aggregate (MTA), has various applications including perforation repair and root-end filling [1, 2]. Recent studies have shown that an ideal root-end filling material should induce regeneration of periradicular tissues, including cementogenesis on the material in addition to providing a proper seal, and should be well tolerated by tissues [3, 4]. The reason behind the use of MTA in such cases is the induction of cementum and bone by MTA in addition to its biocompatibility and the proper seal it provides in the presence of blood and moisture [1, 2]; the exact mechanism of cementogenesis and osteogenesis by MTA is not well elucidated at present [3-6].

One of the hypotheses in relation to the induction of calcification by MTA is the involvement of transforming growth factor-β (TGF-β) family in this process, with an important role in the regulation of a broad range of cellular processes, including growth and differentiation [4-6]. Bone morphogenic proteins (BMPs) are members of TGF-β super family, which can induce calcification in some cells, including pulp stem cells and gingival and periodontal ligament fibroblasts. BMP-2 is one of the mediators involved in the mineralization process induced by MTA [3-5]. Upregulation of this protein has been shown when periodontal ligament fibroblasts, are in direct contact with MTA [5]. In addition, studies have shown the osteoblastic and cementoblastic differentiation of periodontal ligament fibroblasts in the presence of BMP-2 which was induced by MTA [4].

Despite various favorable properties, long setting time and difficult handling are the main disadvantages of MTA [1, 2]. Calcium-enriched mixture (CEM) cement is one of the endodontic biomaterials, which is composed of various calcium ingredients, including its oxide, sulfate, phosphate, carbonate, silicate, hydroxide and chloride [7-9]. Clinical applications of CEM are similar to those of MTA and it has been reported that it has a shorter setting time compared to MTA [7, 8, 10]. Histological evaluations have shown that the inflammatory responses and biologic reactions to CEM and MTA are similar [11-13]. However, contrary to MTA, tissues adjacent to CEM do not exhibit initial necrosis [10, 13].

One of the solutions for improving the properties of MTA, is incorporation of disodium hydrogen phosphate (Na_2_HPO_4_) or DSHP into its composition. Huang *et al.* [14] showed that 15% DSHP solution as a liquid phase, can reduce the setting time of white MTA (WMTA) to 26 min, and a diametral tensile strength of 4.9 MPa at the initial 6-h period can be achieved. They suggested that DSHP solution might be an effective setting accelerator for WMTA. Also DSHP solution as an MTA accelerator, reduces the setting time and maintains the pH value. In addition, Lotfi *et al.* [15] showed that adding DSHP to WMTA creates a more biocompatible material than WMTA alone.

Since no studies have been dedicated to the evaluation of the effect of these new biomaterials on the induction of BMP-2, the present *in vitro* study was undertaken to evaluate the effect of CEM and WMTA+DSHP on the induction of BMP-2 by human gingival fibroblasts (HGF) in comparison to WMTA.

## Methods and Materials

***Cell culture***

After obtaining informed consents, HGFs were obtained from the inflammation-free attached gingiva of premolar teeth from ten healthy individuals undergoing orthodontic extractions. The epithelium of the gingiva was separated from the gingival connective tissue and was immediately placed in Hank’s balanced salt solution (HBSS; Sigma-Aldrich, St Louis, MO, USA), containing penicillin (100 U/mL), streptomycin (100 g/mL), and amphotericin B (5 g/mL). The explants were cut into very small pieces, rinsed in Dulbecco's modified Eagle's medium (DMEM; Gibco Laboratories, Grand Is., NY, USA) twice and left to attach to the bottom of 25-cm^2^ tissue culture flasks (Corning, NY, USA). DMEM, which was supplemented with 10% fetal calf serum, penicillin (100 U/mL) and streptomycin (5 g/mL), was incorporated into the flasks and incubated at 37° C under 95% air/5% CO_2_ condition. Cells isolated from the fourth and fifth passages were used in the present study; because of achieving the best confluency.

***Sample preparation***

The test materials used in this study were ProRoot MTA (Tooth-colored Formula, Dentsply, Tulsa Dental, Tulsa, OK, USA) (WMTA), CEM cement (Yektazist Dandan, Tehran, Iran) and WMTA with DSHP (Na_2_HPO_4_, Merck, Darmstadt, Germany). HGFs (5×104 cells/well) were seeded in 12-well plates (Corning, NY, USA) containing 2 mL of culture medium.

WMTA and CEM were mixed under aseptic conditions according to manufacturer’s instructions. In order to prepare WMTA+DSHP, WMTA was mixed with 2.5 wt% of DSHP. Twelve disks of each material were prepared, measuring 5 mm in diameter and 3 mm in thickness. The disks were wrapped in pieces of moist gauze and incubated for 24 h in a closed container. Then in the relevant plate of each group, WMTA in group 1, CEM in group 2 and WMTA+DSHP in group 3, a disk of biomaterial was placed in each well in proximity with fibroblasts for 72 h, during which the cell culture plates were incubated at 37^°^ C.

***MTT assay***

In order to evaluate the cytotoxic effect of the materials used in the present study, 3-{4,5-dimethylthiazol-2-yl}-2,5-diphenyl tetrazolium bromide colorimetric assay [Mosmann’s tetrazolium toxicity (MTT) assay] was used and was reported as the percentage of viable cells for each well. By this time, 72 h after incubation of the samples, the disks were retrieved from the cell culture media and 2 mL of each cell culture medium was taken from each well for enzyme-linked immunosorbent assay (ELISA). Then MTT solution (5 mg/mL, Sigma Chemical Co., St Louis, MO, USA) was added to each well. Color absorbance was calculated at a wave length of 450 nm.

***ELISA assay***

BMP-2 concentrations were determined by using ELISA kits (Cusabio Biotech Co., LTD., Newark, DE, USA), with the use of human recombinant BMP-2 monoclonal antibodies as standards. In each case, BMP-2 concentration was determined by extrapolating from standard calibration curves. The peroxidase/substrate color change was evaluated with a Synergy HT microplate reader (BioTek Instruments, Winooski, VT, USA) at 450 nm wavelength. The calibration curves, plotted by using regression analysis, were used to read the optical densities of the assays in order to determine BMP-2 concentrations in pg/mL.

**Figure 1:**
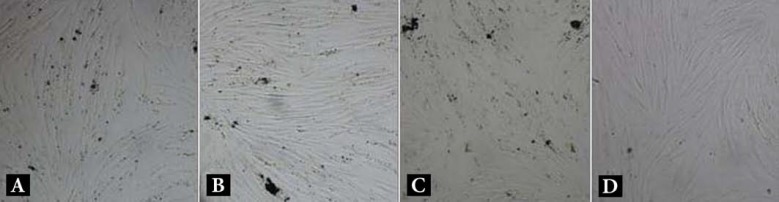
The fibroblast cells in: *A)* close proximity to WMTA+DSHP; *B)* WMTA; *C)* CEM; and *D)* control group, under 40× magnification

**Table 1:** Cell viability and BMP-2 production in study groups

**Groups**	**Cell viability (%)**	**BMP-2 (pg/ml)**
WMTA	65.7	3167±274.46
CEM cement	61.5	2902±240.27
WMTA+DSHP	84.3	1796±836.49
Control	100	2843±325.31

***Statistical analysis***

Statistical analysis was performed by the one-way ANOVA test and because of abnormality of variables it was followed by the post hoc Games-Howell test for BMP-2 and one-way ANOVA, followed by post hoc Tukey’s test for MTT assay. Statistical significance was set at 0.05. Statistical analysis was carried out by SPSS software (SPSS version 18.0, SPSS, Chicago, IL, USA).

## Results

***MTT assay***

The highest cell viability (100%) was achieved in the control group, with significant differences from the other groups (*P*<0.05). Cell viability in the presence of DSHP was significantly higher (84.3%) than that in groups 1 and 2 (*P*<0.05). CEM (61.5%) and WMTA (65.7%) did not exhibit any significant differences in cell viability (*P*=0.08). Figure 1 demonstrates the cells in proximity of the test materials.

***BMP-2 assay***

The highest and lowest production rates of protein were observed in the WMTA (3167±274.46) and WMTA+DSHP (1796±836.49) groups, in order of appearance. BMP-2 concentrations were 2843±325.31 and 2902±240.27 pg/mL in the control and CEM groups, respectively. One-way ANOVA showed statistically significant differences between the groups (*P*=0.01). Two-by-two comparison of the groups with Games-Howell test showed no significant differences between the CEM and WMTA groups regarding the amount of produced protein (*P*=0.08). In addition, there were no significant differences between these groups and the control group (*P*>0.05). The amount of protein produced in the WMTA+DSHP group was significantly lower than that produced in the other groups (*P*<0.05) (Table 1).

## Discussion

The ability to induce tissue regeneration is a favorable characteristic for any material which is placed in close proximity to vital tissues. MTA is a notable example because it is used for the repair of root perforations and as a root-end filling material [1, 2]. MTA induces calcification in the cells it comes into contact with, including pulp and periodontal ligament cells [4-6, 15-18]. As the members of the TGF-β family, the activity of BMPs has been reported as the molecular basis for this process [4, 19, 20].

The results of the present study showed that 72 h after exposure of HGFs to WMTA, there was an increase in the induction of BMP-2, with no significant differences from the control group. It is hypothesized that this inductive property of MTA is due to the release of calcium from MTA, which by itself induces production of BMP-2 [4, 5, 21]. BMP-2 is bound to serine/thyrosine kinase receptors and produces a cascade of signals to regulate cell functions such as osteogenesis/cementogenesis [16]. In other words, BMP-2 induces the osteoblastic/cementoblastic differentiation of cells [3, 6, 17]. It has been demonstrated that gingival fibroblasts exhibit behaviors similar to those of periodontal ligament fibroblasts and express genes that are indicative of cementogenesis and osteogenesis activities [5].

CEM can release calcium as well [7], and is used in a similar manner to MTA as a root-end filling material and for furcation perforation repair because it can induce precipitation of cementum [8, 12, 22, 23]. Torabinejad reported that precipitation of cementum at close proximity to MTA depends on several factors: creation of an alkaline environment by the material and the sealing ability and biocompatibility of the material [24]. Studies have shown that the two latter properties are similar in CEM and MTA; however, CEM has a higher alkaline property compared to MTA [7, 21].

In the present study after the cells were incubated with the materials under study the viability of the cells was evaluated by MTT assay, which is an easy technique with high sensitivity for the evaluation of cytotoxicity [5]. The cells reacted to WMTA and CEM in a relatively similar manner with no significant differences in cell viability. Lack of differences in the amount of protein produced in WMTA and CEM groups compared to the control group might be attributed to the initial toxicity of these materials, which results in the lysis of a number of cells in the culture media [21]; on the other hand, the duration of exposure of the cells to the materials is short. In fact, the cells have a limited time interval (72 h) to express the necessary genes for producing protein.

One of the techniques to improve the properties of MTA is to incorporate DSHP into its composition to decrease its setting time [14, 15]. Huange *et al*. [14] showed that the resultant material has comparable *in vitro* biocompatibility. Lotfi *et al.* [15] showed that incorporation of 2.5 wt% of DSHP to MTA, improves its biocompatibility. Based on the results of MTT assay, cell viability in the presence of DSHP was significantly higher compared to other study groups, consistent with the results of previous studies. In the present study the amount of BMP-2 produced in the WMTA+DSHP group was less than that of other groups. When DSHP is added to MTA, disodium reacts with calcium hydroxide, thus reducing the amount of calcium hydroxide released into the environment [15]. The results of the present study, regarding the amount of BMP-2 production in presence of DSHP being insignificant, can be justified by the mechanism of setting and the mechanism explained above in relation to the effect of calcium on induction of BMP-2 production.

The present study was carried out *in vitro*; thus it was not possible to evaluate the possible effect of refreshing the tissue fluid and also division and replacement of the cells, which have a role in neutralization of the toxic effects of materials. Therefore, performing *in vivo* studies with longer time intervals is suggested to better simulate the tissue environmental conditions and sustained release of calcium from these biomaterials.

## Conclusion

BMP-2 inducing ability of CEM cement or WMTA in human gingival fibroblasts was similar and they did not have a significant difference; however the amount of BMP-2 production decreased when DSHP was added to WMTA. 
